# Pressure-Less Acoustic Immittance-Derived Middle Ear Resonance Frequency: Repeatability and Comparison with Multifrequency Tympanometry

**DOI:** 10.3390/audiolres16040098

**Published:** 2026-06-30

**Authors:** Zsuzsanna Piros, Szilárd Gyula Rezes, Rita Kiss, Beáta Lelesz, Gyula Ujlaki, Béla Juhász, Zoltán Szilvássy

**Affiliations:** 1 Szilvássy Judit Audiology Centre, Department of Otorhinolaryngology and Head and Neck Surgery, University of Debrecen Clinical Centre, Nagyerdei St. 98, H-4032 Debrecen, Hungary; rezes.szilard@med.unideb.hu; 2 Doctoral School of Nutrition and Food Sciences, University of Debrecen, H-4032 Debrecen, Hungary; juhasz.bela@med.unideb.hu (B.J.); szilvassy.zoltan@med.unideb.hu (Z.S.); 3 Department of Pharmacology and Pharmacotherapy, Faculty of Medicine, University of Debrecen, Nagyerdei St. 98, H-4032 Debrecen, Hungary; kiss.rita@med.unideb.hu (R.K.); lelesz.beata@med.unideb.hu (B.L.); ujlaki.gyula@med.unideb.hu (G.U.)

**Keywords:** middle ear resonance frequency, multifrequency tympanometry, pressure-less acoustic immittance, middle ear mechanics, repeatability, reliability, Bland–Altman analysis, intraclass correlation coefficient, middle ear diagnostics, methodological comparison study

## Abstract

**Background/Objectives**: This study evaluated the intra-session repeatability, reliability, and clinical consistency of resonance frequency derived from pressure-less acoustic immittance (PLAI), and compared the results with those obtained using conventional multifrequency tympanometry under identical clinical conditions. **Methods**: In this pilot methodological comparison study, resonance frequency (RF) measurements were obtained in 15 subjects (30 ears) using multifrequency tympanometry (MFT) and pressure-less acoustic immittance (PLAI). Three consecutive measurements were performed for each ear using both methods. Agreement between methods was evaluated using Bland–Altman analysis. Within-method repeatability was assessed using range, coefficient of variation, repeatability coefficient, and intraclass correlation coefficients. **Results**: PLAI demonstrated lower intra-session variability than MFT. The mean within-series SD was 96.6 Hz for MFT and 10.5 Hz for PLAI. The coefficient of variation was 9.3% for MFT and 3.2% for PLAI, while intraclass correlation coefficients were 0.60 and 0.83, respectively. The repeatability coefficient was ±367 Hz for MFT and ±47 Hz for PLAI. Bland–Altman analysis revealed a systematic difference between the two methods, with a mean bias of 580.7 Hz and 95% limits of agreement between 247.5 Hz and 913.9 Hz, indicating that the two methods are not interchangeable. **Conclusions**: PLAI demonstrated higher repeatability than MFT. Its lower variability may support a more consistent clinical interpretation; PLAI-derived RF may therefore represent a stable method-specific parameter for middle ear assessment. However, RF values should be interpreted within method-specific frameworks.

## 1. Introduction

The human middle ear functions as a coupled mechano-acoustic impedance matching system that transfers sound energy from air to the fluid-filled cochlea. Its dynamic behavior is determined by the interaction of mass, stiffness, and damping components. Resonance frequency reflects the balance between these components and therefore provides information about the mechanical state of the middle ear system.

The resonance frequency (RF) of the middle ear is a clinically relevant parameter that reflects the mechanical state of the sound conduction system and may indicate pathological changes earlier than several conventional diagnostic methods. For this reason, RF has considerable diagnostic value in conditions affecting the middle ear, such as otosclerosis and other conductive pathologies, and may also be influenced by inner ear and systemic disorders [1,2,3,4,5,6]. In linear mechanical systems, resonance frequency is defined as the frequency at which the reactive components of the system cancel each other out, resulting in zero net reactance and maximal energy transfer. However, the middle ear is a complex biological system and its resonance frequency cannot be measured directly in vivo. Instead, RF can only be estimated indirectly using acoustic or immittance-based methods [7,8].

Despite the availability of otoscopy, pure-tone audiometry, and objective middle-ear assessment techniques, the evaluation of middle-ear function remains challenging. Individual diagnostic methods provide complementary rather than definitive information, and discrepancies between clinical findings, audiometric results, and tympanometric measurements are not uncommon. In such situations, resonance frequency assessment may provide additional information regarding the mechanical status of the middle ear and help reduce diagnostic uncertainty [6,9,10].

Multifrequency tympanometry (MFT) has long been considered the clinical standard for estimating middle-ear resonance frequency. The method evaluates acoustic admittance across a range of probe frequencies while applying controlled pressure variations within the external auditory canal. Resonance frequency is determined from frequency-dependent changes in admittance, susceptance, and conductance characteristics and corresponds to the point at which stiffness- and mass-related contributions become balanced. In modern clinical systems, RF is identified automatically by device-specific algorithms and reported as a single resonance-frequency estimate. Normal adult RF values generally range between 800 and 1200 Hz. However, RF determination may be influenced by measurement resolution, damping characteristics, and interpretation of tympanometric morphology, potentially affecting reproducibility and clinical consistency [6,9,10,11,12].

A major limitation of both conventional tympanometry and MFT is the requirement for controlled pressure changes within the external auditory canal. This requirement may influence the mechanical properties being measured and may limit applicability in several clinically important situations, including tympanic membrane perforation, ventilation tubes, postoperative middle-ear conditions, or cases where an airtight seal cannot be achieved. Importantly, these are often the clinical scenarios in which objective assessment of middle-ear mechanics would be of greatest diagnostic value [6,9,12].

Alternative approaches to middle-ear assessment have therefore been developed, including wideband acoustic immittance (WAI) and wideband tympanometry (WBT). Although these terms are sometimes used interchangeably, they do not represent identical measurement approaches. WAI is typically performed under ambient pressure conditions and evaluates sound energy transfer across a broad frequency range without the application of ear-canal pressurisation. In contrast, WBT combines wideband acoustic measurements with tympanometric pressure sweeps, allowing assessment of middle-ear function across different ear-canal pressure conditions [13,14].

Both methods provide substantially more frequency-specific information than conventional tympanometry and have demonstrated diagnostic value in a variety of middle-ear disorders. However, despite these advantages, the widespread implementation of WAI/WBT in routine clinical practice has remained limited. The interpretation of the large amount of generated frequency-dependent data may be challenging, normative databases are still evolving, and clinical decision-making often relies on multiple parameters rather than a single easily interpretable outcome measure [13,14,15].

Pressure-Less Acoustic Immittance (PLAI) was subsequently introduced as a pressure-free approach to middle-ear assessment. Similar to WAI/WBT, its primary purpose was not resonance-frequency estimation but the evaluation of middle-ear function without the need for ear-canal pressurisation. By eliminating pressure changes within the external auditory canal, PLAI was designed to overcome several practical limitations of conventional immittance measurements while providing clinically interpretable parameters suitable for routine diagnostic use [13,14,15].

PLAI is the proprietary methodology implemented in the med·wave^®^ system (Neuranix Srl, Trieste, Italy) and should not be considered synonymous with conventional wideband acoustic immittance (WAI) or wideband acoustic absorbance (WBA). The system uses a broadband chirp stimulus (100–3000 Hz) and analyses the reflected acoustic energy without applying a pressure sweep. Based on the frequency-dependent behaviour of admittance-related parameters derived from the reflected signal, the device automatically estimates resonance frequency (Fr) defined as the frequency corresponding to the maximum of the admittance curve, and provides additional output parameters including peak admittance (P) [16].

Using microelectromechanical systems (MEMS) technology, PLAI enables simultaneous measurement of acoustic pressure and particle velocity, allowing multiparametric evaluation of middle ear mechanics under physiological conditions. In addition to categorical classification of middle ear status, the system also provides a resonance frequency value during measurement [5,13,15,16].

Importantly, the PLAI-derived resonance frequency parameter was not the primary target of the technology’s development. Rather, it represents an additional output generated by the system, whose repeatability, reliability, clinical consistency, and potential diagnostic utility have not yet been systematically evaluated [17]. Although PLAI has been validated as a diagnostic tool for middle-ear assessment, the reliability and clinical interpretability of the PLAI-derived RF parameter have not been sufficiently investigated.

The primary objective of the study was to evaluate the intra-session repeatability, reliability, and clinical consistency of PLAI-derived resonance-frequency measurements. As MFT is currently the most widely used clinical method for estimating middle-ear resonance frequency, PLAI-derived RF values were additionally compared with MFT-derived RF measurements obtained under identical clinical conditions.

## 2. Materials and Methods

### 2.1. Study Design

The primary aim of the study was to evaluate the intra-session repeatability, reliability, and clinical consistency of PLAI-derived resonance-frequency measurements. Measurements obtained by conventional multifrequency tympanometry were analysed in parallel to provide a clinical reference for comparison under identical testing conditions. Repeatability was assessed by performing three consecutive RF measurements under identical conditions in the same ear, using the same device and examiner within a single session. To this end, the stability of RF values was assessed using series of three consecutive measurements.

The aim was therefore to assess short-term intra-session repeatability under repeated real-world clinical measurement conditions.

### 2.2. Participants

Patients presented to the Szilvássy Judit Audiology Centre (Department of Otolaryngology and Head and Neck Surgery, University of Debrecen Clinical Centre) were included in the study. The inclusion criterion was the written consent required for voluntary participation in the study. Exclusion criteria included pathological conditions of the middle ear and tympanic membrane, conductive hearing loss, or suspicion thereof. Based on these criteria, 15 subjects (5 males, 10 females) were included in the study, aged 12–58 years (mean age 44.8 ± 12.8 years).

This study was conducted in accordance with the Declaration of Helsinki and was approved by the Regional Institutional Research Ethics Committee of the Clinical Centre, University of Debrecen (approval number: 7337-2025 RKEB). All participants provided written informed consent prior to participation.

### 2.3. Audiological Assessments

In all subjects, microotoscopy performed during the ENT examination revealed a closed tympanic cavity and an intact tympanic membrane. Hearing status was assessed using pure-tone audiometry. Normal hearing was defined as pure-tone thresholds not exceeding 20 dB HL between 250 and 2000 Hz and 25 dB HL at all other tested frequencies.

Middle ear status was assessed by tympanometry. Tympanometry was performed with GSI Tympstar Pro 2 (Grason-Stadler Inc., Eden Prairie, MN, USA), running App Version 2.1.1 (Build 3). Tympanometric classification was based on the normative criteria implemented in the GSI TympStar Pro 2 system, according to the manufacturer’s normative ranges for tympanometric peak pressure and static admittance, as provided by the device software. Only ears fulfilling the manufacturer-defined criteria for a Type A tympanogram were included in the study.

### 2.4. Multifrequency Tympanometry

Multifrequency tympanometry measurements were performed using the GSI TympStar Pro 2 system running App Version 2.1.1 (Build 3).

The TympStar Pro system performs multifrequency tympanometry using a frequency-scanning protocol (Grason-Stadler Inc., Eden Prairie, MN, USA) across probe frequencies ranging from 200 to 2000 Hz with 50 Hz resolution steps. Resonance frequency is automatically identified by the device software based on the frequency-dependent behaviour of admittance-related components and reported as a single RF value.

The RF values used in the present study were the standard automated outputs generated by the instrument and were not manually selected or interpreted by the examiner. For conventional multifrequency tympanometry (MFT), resonance frequency values between 900 and 1200 Hz were considered within the normal range. Resonance frequencies above this range were interpreted as indicating increased stiffness of the middle ear system, whereas lower values indicated increased flaccidity of the sound conduction system.

### 2.5. Pressure-Less Acoustic Immittance

PLAI measurements were performed using the med·wave^®^ system (Neuranix Srl, Trieste, Italy) running software version V4.01.03.

The PLAI-derived RF value was obtained directly from the med·wave^®^ system output and should be interpreted as a device-specific resonance-related parameter. For pressure-less acoustic immittance measurements (PLAI), normative values were determined according to the latest literature. In healthy ears, the mean resonant frequency was 503.3 Hz ± 140.6 Hz, with a range of approximately 285.3 to 1119 Hz. Under normal conditions, the mean peak admittance values were 1.50 × 10^−2^ mmho, with a range of 0.49–3.75 × 10^−2^ mmho.

The instruments were used with valid manufacturer-recommended settings, accessories, and calibrations. For each participant, both ears were examined using both measurement methods: multifrequency tympanometry and pressure-less acoustic immittance measurement.

### 2.6. Measurement Protocol

All measurements on a given subject were performed by the same examiner in each case to minimize the possibility of inter-examiner variability. Between repeated measurements, the probe or ear tip was removed, the participant rested for at least one minute, probe fitting was repeated, and the next measurement was started as a new independent clinical measurement. All measurements were performed consecutively in both ears on the same day in the quiet environment of the audiometry laboratory. The order of the measurements was not random, as pressure changes during the MFT could temporarily affect the mechanics of the middle ear; in all cases, the MFT measurement was performed first, followed by the PLAI measurement.

These repeated measurements were used to assess intra-session repeatability for each method separately. The series of three consecutive measurements was performed to assess the agreement between the two methods, as well as the intra-session reliability, intra-session repeatability, and clinical consistency of each method.

### 2.7. Data Processing and Statistical Analysis

Within each measurement series obtained from a single ear, the following derived data (descriptive statistics) were calculated from the three measurement results: range, mean, and standard deviation. The agreement between methods was assessed using Bland–Altman analysis, as recommended for comparison of non-equivalent measurement techniques. The repeatability within each series was characterised by the range (R), the coefficient of variation (CV), and the coefficient of repeatability (RC). Reliability was assessed using the intraclass correlation coefficient (ICC, two-way mixed-effects model, absolute agreement, single measurement) [8]. Clinical consistency of the measurements was assessed using method-specific reference ranges based on normative data previously published. This determined whether repeated measurements within the same measurement series resulted in concordant or discordant diagnostic classifications (normal vs. pathological middle ear condition).

All statistical analyses were performed using IBM SPSS Statistics version 31.0 (IBM Corp., Armonk, NY, USA).

## 3. Results

Applying the above inclusion and exclusion criteria, 15 subjects (5 males, 10 females) were included in the study, representing a total of 30 ears examined. Micro-otoscopy revealed an intact tympanic membrane in all ears, and tympanometry recorded a type-A tympanogram in all cases.

Table 1 summarizing the demographic and clinical characteristics of the participants, including age, sex distribution, and relevant audiological information.

**Figure 1:**
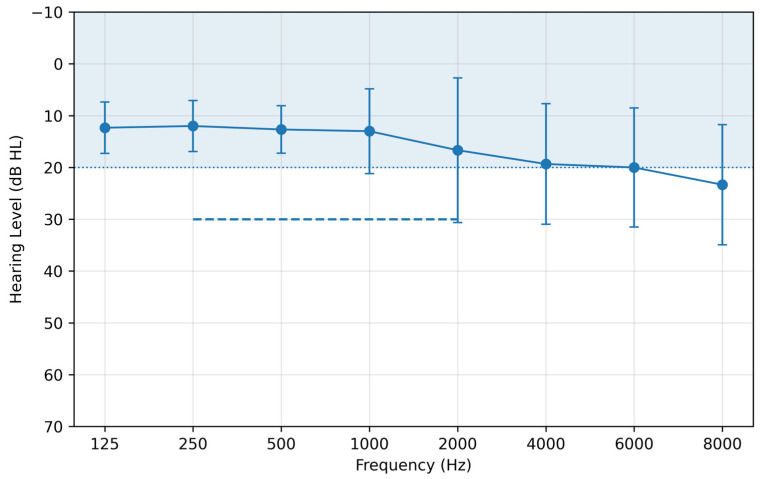
Mean air-conduction pure-tone thresholds of the study population (n = 15). Error bars represent standard deviation. The shaded area indicates the normal hearing range (≤20 dB HL). The short dashed line indicates the social/speech-frequency hearing threshold (30 dB HL between 500 and 1000 Hz).

Based on the results of pure tone audiometry (PTA), thirteen subjects had normal hearing, while two subjects had sensorineural hearing loss without a conductive component (bone–air gap). Figure 1 shows the mean pure-tone audiogram of the study population together with standard deviation values. All included ears demonstrated Type A tympanograms and no evidence of conductive hearing loss (air–bone gap = 0).

### 3.1. Agreement Between Methods

**Figure 2:**
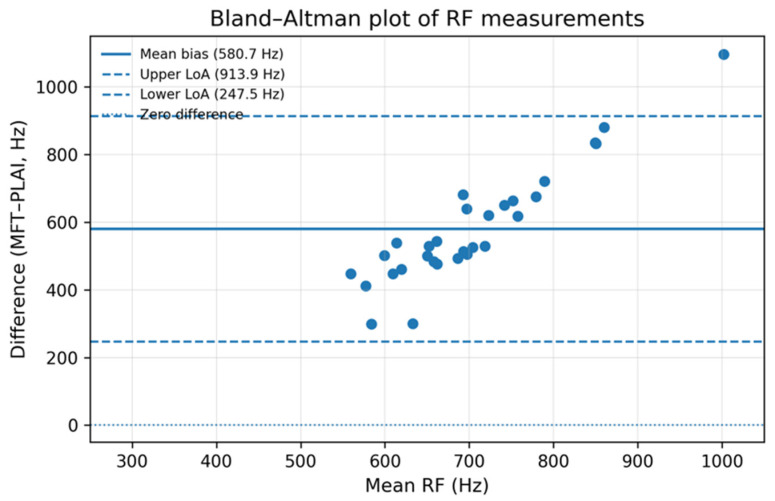
Bland–Altman plot of RF values obtained with MFT and PLAI. Differences are plotted against the mean RF. The solid line indicates the mean bias (580.7 Hz), dashed lines indicate the 95% limits of agreement (247.5–913.9 Hz), and the dotted line represents zero difference.

The agreement between the two measurement techniques was first evaluated using Bland–Altman analysis. This demonstrated a substantial systematic bias between the two methods (Figure 2). The mean difference (MFT–PLAI) was 580.7 Hz, with 95% limits of agreement ranging from 247.5 Hz to 913.9 Hz, indicating a substantial systematic difference between the two methods. These data are shown in Figure 2. As no agreement was observed between the methods, further analysis focused on within-method repeatability.

### 3.2. Repeatability Analysis

PLAI demonstrated superior repeatability over MFT, with within-series standard deviation and repeatability coefficients (RCs) nearly tenfold lower (Table 2).

**Figure 3:**
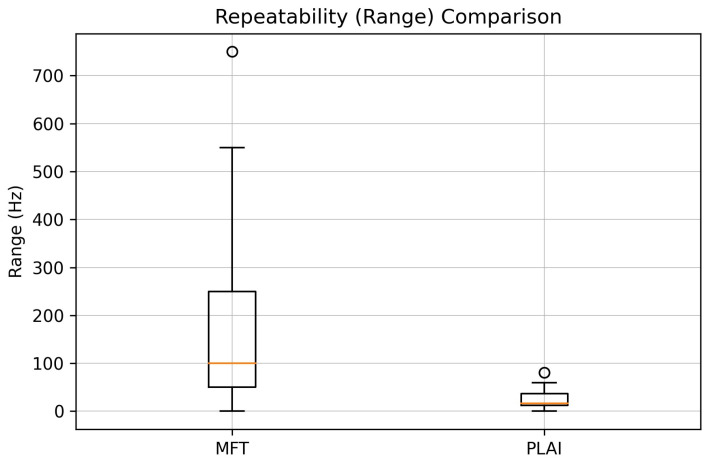
Distribution of within-series ranges for MFT and PLAI measurements. PLAI demonstrated substantially lower dispersion, indicating superior intra-session repeatability.

Figure 3 shows the distribution of the ranges within the series (R) for MFT and PLAI measurements. The substantially narrower distribution observed for PLAI indicates lower intra-session variability and better repeatability compared to MFT.

### 3.3. Reliability

Reliability analysis showed higher intraclass correlation coefficients for PLAI (ICC = 0.83) compared to MFT (ICC = 0.60), indicating superior measurement reliability. Although MFT showed only moderate reliability, PLAI reached a level consistent with good reliability, supporting its suitability for repeated RF measurements.

### 3.4. Clinical Consistency Analysis

The clinical consistency analysis also highlighted the differences between the two methods. With MFT, clinically discordant results were observed in 10 of 30 ears (33.3%), indicating that repeated measurements within the same session could lead to inconsistent diagnostic classification. In contrast, PLAI demonstrated complete clinical concordance across repeated measurements, with only minor borderline variations that did not affect overall classification. These findings suggest that the higher variability in MFT may compromise clinical interpretability, whereas PLAI provides more stable and clinically consistent results.

## 4. Discussion

The present study demonstrates that pressure-less acoustic immittance (PLAI) provides substantially better intra-session repeatability in estimating the resonance frequency of the middle ear compared to multifrequency tympanometry (MFT). Importantly, assessment of agreement between PLAI-derived and MFT-derived RF values was not the primary objective of the study. Rather, Bland–Altman analysis was performed as an exploratory secondary analysis to determine whether the RF estimates obtained by the two fundamentally different measurement approaches could be considered interchangeable. Therefore, the observed lack of agreement should not be interpreted as evidence of the inferior performance of either method. Rather, it reflects the fact that MFT-derived and PLAI-derived RF values represent different resonance-related descriptors of middle-ear mechanics.

This observation is biologically plausible. MFT and PLAI do not measure the same physical phenomenon using different instruments; rather, they estimate resonance-related behaviour through fundamentally different measurement principles. MFT derives RF from pressure-dependent admittance characteristics, whereas PLAI estimates RF from broadband reflected acoustic energy obtained under ambient pressure conditions. Consequently, systematic differences between the RF values generated by the two methods should be expected.

At the same time, the observed systematic difference between the two methods indicates that they should not be considered interchangeable. However, from a clinical perspective, the two methods may provide complementary information regarding middle-ear mechanics and therefore help reduce diagnostic uncertainty in challenging cases.

This finding is consistent with the earlier multifrequency tympanometry literature, which demonstrated that the method used to estimate resonance frequency has a significant impact on the measured values, and comparisons across different measurement techniques are therefore not valid [10]. In this context, the discrepancy observed between MFT-derived RF and PLAI-derived RF in the present study is not unexpected but rather reflects the inherent methodological differences between the two approaches. One possible explanation for this difference is that the middle ear represents a complex, multi-component mechanical system, in which the measured resonance frequency reflects the combined behaviour of the tympanic membrane, ossicular chain, ligament system, and cochlear loading. MFT determines RF based on frequency-dependent acoustic admittance under varying ear canal pressure, identifying the point where mass and stiffness components are balanced. In contrast, PLAI operates under atmospheric pressure conditions and evaluates broadband acoustic response characteristics using simultaneous measurements of acoustic pressure and particle velocity. As a result, PLAI-derived RF reflects the frequency of maximal energy transfer or phase transition rather than the zero-crossing of susceptance, leading to systematically different RF values. Compared with MFT, PLAI demonstrated consistently lower within-series range, lower standard deviation, and lower variance, indicating superior intra-session repeatability and reduced measurement variability under identical clinical conditions. These findings support the stability of PLAI-derived RF measurements and further support its potential clinical applicability as a repeatable method-specific parameter for middle-ear assessment. While this methodological variability limits direct comparability, the present results highlight a potentially important advantage of PLAI: improved within-method stability. Previous studies have reported only moderate reliability of RF measurements obtained with MFT [12], whereas recent PLAI studies describe the method as a pressure-free, non-invasive approach for middle-ear assessment under physiological conditions. This may be particularly advantageous in clinical situations where conventional tympanometry is technically challenging, such as in infants or under post-tympanoplastic conditions.

Based on these findings, PLAI-derived RF should be interpreted as a method-specific parameter rather than as a direct equivalent of MFT-derived RF. The clinical value of PLAI lies not in reproducing MFT-based RF values but in providing a stable and physiologically relevant measurement that may support the assessment of the middle ear within its own diagnostic framework.

The present study has several limitations. First, the sample size was relatively small and consisted predominantly of normal ears, limiting the generalisability of the findings. Second, both ears from the same participant were included in the analysis and treated as independent observations. Although this approach increased the number of available measurements, it may have introduced within-subject correlation and therefore influenced some statistical estimates. Third, all measurements were performed during a single session and therefore reflect intra-session rather than long-term repeatability. Fourth, the order of measurements was not randomised. MFT was always performed before PLAI because tympanometric pressure changes may temporarily influence middle-ear mechanics and could potentially affect subsequent measurements. Consequently, an order effect cannot be completely excluded. Finally, the present study should be regarded as exploratory and hypothesis-generating. Larger studies, including pathological middle-ear conditions and independent validation cohorts, will be required before definitive clinical conclusions can be drawn.

## 5. Conclusions

PLAI-derived RF appears to be a stable and reproducible parameter; however, its clinical interpretation should be performed within the methodological framework of PLAI and not directly compared with MFT-derived RF.

## Figures and Tables

**Table 1:** Demographic and clinical characteristics of the participants.

Characteristic	Value
Participants (*n*)	15
Ears analysed (*n*)	30
Female, *n* (%)	10 (66.7%)
Male, *n* (%)	5 (33.3%)
Age, mean ± SD (years)	44.9 ± 12.8
Age range (years)	12–58
Type A tympanogram	30/30 ears
Sensorineural hearing loss	2/15 participants
Air–bone gap	0/30 ears

**Table 2:** Repeatability analysis of RF measurement techniques (MFT and PLAI). Range (R) was defined as the difference between the maximum and minimum RF values obtained within each series of three repeated measurements. Mean range represents the arithmetic mean of these within-series range values across all analysed ears. Range parameters do not represent the distribution of RF values across the study population.

Parameter	MFT	PLAI
Number of ears	30	30
Mean RF (Hz)	992.2	408.97
Within-series range (R = max(x_i_) − min(x_i_))	0–750	0.61–80.75
Mean of within-series range (Hz)	170	24.96
Variance of within-series range	30,620.69	365.70
Mean SD within-series (Hz)	96.55	10.46
Variance of SD	9315.05	109.46
Grand mean (Hz)	992.8	412.1
Coefficient of variation (CV)	9.3%	3.2%
Repeatability coefficient (RC)	±367	±47

## Data Availability

Data supporting the findings of this study are available from the corresponding author upon reasonable request.

## Abbreviations

The following abbreviations are used in this manuscript:

RF	Resonance Frequency
MFT	Multifrequency tympanometry
PLAI	Pressure-less acoustic immittance
MEMS	Microelectromechanical systems
R	Range
CV	Coefficient of variation
RC	Coefficient of repeatability
ICC	Intraclass correlation coefficient
